# Unconscious Memory in Disease

**Published:** 1885-12

**Authors:** 


*Unconscious Memory in Disease.* By CHARLES CREIGHTON, M.D. Pp. 212. London: H. K. LEWIS. 1885.

This is undoubtedly a powerful and remarkable book. We were fully prepared for abundant evidences of philosophic breadth and culture in anything that falls from the pen of Dr. Creighton relating to pathology; but we confess we were somewhat surprised to find proofs of at least equal capacity in dealing with matters relating to practical medicine. No doubt pathology is the *scientia scientiarum* for all medicine; a profound knowledge of pathology renders all medicine easy. But, in this country at least, we have become so familiar with the mere mechanician, the cutter of sections and the mounter of slides, as the only exponent of pathology, that we have some difficulty in realising the fact that a learned pathologist may be at the same time a high-class physician.

It is impossible to give, in the space at our disposal, an adequate idea of the purpose and aims of Dr. Creighton's work. It is mainly an estimate of the influence of habit and memory, physiologically, pathologically, and practically considered, as continuing disease. His thesis is supported by an abundance of illustration, drawn from the most varied sources, and the propositions are knit together by conclusions drawn from practice. If we were surprised at the number of facts which the author has been able to concentrate on his subject, we were charmed with the manner in which he has manipulated them, and logically evolved their meaning. Even if we were prepared to dispute any of his conclusions, we could not fail to admit the masterly handling of logical methods whereby he reaches them. In language Dr. Creighton has here excelled himself. Possibly this is the first opportunity which his practical labours have afforded him of showing his powers in exposition; certainly this opportunity has proved that Dr. Creighton is second to no living medical author as a writer of the English language.

We confess to having laid down the work with something like a glow of enthusiasm in our somewhat nauseated intellect. It has been the case with one other modern work besides this, that the reviewer could read nothing else till he had finished it—Hilton's *Rest and Pain.* With Hilton's classical work it has little in common beyond this, that it deals with one dominant idea, and that it illustrates and expands this idea with a fertility and breadth that had never before been excelled, or even attempted. We would even go so far as to say that in logic and language Creighton has surpassed Hilton. Our readers will judge for themselves.

